# Gaining insight on mitigation of rubeosis iridis by UPARANT in a mouse model associated with proliferative retinopathy

**DOI:** 10.1007/s00109-020-01979-8

**Published:** 2020-09-17

**Authors:** Filippo Locri, Noemi A. Pesce, Monica Aronsson, Maurizio Cammalleri, Mario De Rosa, Vincenzo Pavone, Paola Bagnoli, Anders Kvanta, Massimo Dal Monte, Helder André

**Affiliations:** 1 Department of Clinical Neuroscience, Division of Eye and Vision, St Erik Eye Hospital, Karolinska Institutet, Polhemsgatan 50, 112 82 Stockholm, Sweden; 2 Department of Biology, University of Pisa, Pisa, Italy; 3 Department of Experimental Medicine, Second University of Naples, Naples, Italy; 4 Department of Chemical Sciences, University of Naples Federico II, Naples, Italy

**Keywords:** Rubeosis iridis, Proliferative retinopathy, Inflammation, Antiangiogenic drug, UPARANT

## Abstract

**Abstract:**

Proliferative retinopathies (PR) lead to an increase in neovascularization and inflammation factors, at times culminating in pathologic rubeosis iridis (RI). In mice, uveal puncture combined with injection of hypoxia-conditioned media mimics RI associated with proliferative retinopathies. Here, we investigated the effects of the urokinase plasminogen activator receptor (uPAR) antagonist—UPARANT—on the angiogenic and inflammatory processes that are dysregulated in this model. In addition, the effects of UPARANT were compared with those of anti-vascular endothelial growth factor (VEGF) therapies. Administration of UPARANT promptly decreased iris vasculature, while anti-VEGF effects were slower and less pronounced. Immunoblot and qPCR analysis suggested that UPARANT acts predominantly by reducing the upregulated inflammatory and extracellular matrix degradation responses. UPARANT appears to be more effective in comparison to anti-VEGF in the treatment of RI associated with PR in the murine model, by modulating multiple uPAR-associated signaling pathways. Furthermore, UPARANT effectiveness was maintained when systemically administered, which could open to novel improved therapies for proliferative ocular diseases, particularly those associated with PR.

**Key messages:**

• Further evidence of UPARANT effectiveness in normalizing pathological iris neovascularization.

• Both systemic and local administration of UPARANT reduce iris neovascularization in a model associated with proliferative retinopathies.

• In the mouse models of rubeosis iridis associated with proliferative retinopathy, UPARANT displays stronger effects when compared with anti-vascular endothelial growth factor regimen.

**Electronic supplementary material:**

The online version of this article (10.1007/s00109-020-01979-8) contains supplementary material, which is available to authorized users.

## Introduction

In the eye, vascular networks are essential to provide oxygen and nutrients to the cells. The uvea, comprising the choroid, the ciliary body, and the iris, represents the most vascularized ocular tissue. The iris vasculature is characterized by abundant arterio-venous anastomoses, and supply nutrients to the anterior chamber of the eye, including the avascular trabecular meshwork, cornea, and lens. Furthermore, the iris vascular network represents a major source of oxygen in the aqueous humor [1]. During embryonic development and in adult life, angiogenesis processes lead to the formation of new blood vessels from pre-existing vasculature.

Angiogenesis is finely regulated by several angiogenic stimulators and inhibitors. The vascular endothelial growth factor (VEGF) is the most canonical angiogenic mediator, yet other factors, such as the plasminogen-activator system and inflammatory factors, play an essential role in neoangiogenesis. A molecular imbalance between angiogenic inducers and inhibitors results in neoangiogenesis, a common denominator in several ophthalmic diseases that can lead to blindness [2]. In many proliferative ocular diseases, the neovascularization is located in the retina; however, some advanced stages of proliferative diabetic retinopathy (PDR) and central retinal venous occlusion (CRVO) can trigger pathologic iris neovascularization, clinically determined rubeosis iridis (RI) [3, 4]. In proliferative retinopathies (PR), the increase in vitreal neoangiogenic factors induces iris neovascularization [5]. The proliferation of new blood vessels, along the surface of the iris, can lead to obstruction of the aqueous humor flow, increased intraocular pressure, and finally neovascular glaucoma (NVG) [6].

Anti-VEGF agents are effective in reducing RI; nevertheless, there are some concerns, since the effects are limited [7–10]. The small tetrapeptide UPARANT has been shown to reduce endothelial cell proliferation, motility and tube formation by interfering between the complex crosstalk interaction of plasmin activity, and the urokinase-type plasminogen activator receptor (uPAR) and formyl peptide receptors (FPRs) [11–14]. uPAR and its ligand, the urokinase-type plasminogen activator (uPA), have an important role in angiogenesis and inflammation. uPAR-uPA system regulates the degradation of specific components of the basement membrane and extracellular matrix, processes essential for the penetration of the capillary basement membrane by sprouting endothelial cells. UPARANT has been tested in several rodent models of PR [15–18]; the pharmacological administration of UPARANT has demonstrated effectiveness in reducing angiogenesis and ameliorating visual dysfunction. Previously, in a mouse model of induced RI [19, 20], we have demonstrated the effectiveness of UPARANT in mitigating RI [21]. In this model, the major drive of iris neovascularization is wound healing responses, with increased expression of the plasminogen-activator and inflammation systems, and has the advantage of allowing for direct, noninvasive quantification of the iris vasculature in vivo.

In the present study, we have extended the puncture-induced RI model and associated it with PR (RI-PR) by co-injection of hypoxia-conditioned medium to mimic the increase in proangiogenic pressure from the posterior compartment of the eye. UPARANT efficacy in counteracting the exacerbated iris neovascular response characteristic of the RI-PR model was assayed and compared with a mouse equivalent of aflibercept, an anti-VEGF drug in clinical use. Moreover, mitigation of iris vascular responses by UPARANT in the RI-PR was determined both by intravitreal and subcutaneous administrations.

## Materials and methods

### Pharmacological treatments

UPARANT (World Health Organization international non-proprietary name Cenupatide; CAS number: 1006388-38-0) [11, 22] was solubilized in sterile phosphate buffered saline (PBS; ThermoFisher Scientific Inc., Waltham, MA, USA) as a succinate salt at 10 mg/mL (intravitreal administration) and 20 mg/kg (subcutaneous administration). These concentrations correspond respectively to 7.6 mg/mL and 15.2 mg/kg of active pharmaceutical ingredient, as previously reported [16]. The mouse equivalent of aflibercept, a VEGF-receptor (VEGFR)1 ligand-binding domain/Ig Fc chimeric protein (referred to as VEGFR1 chimera), was purchased from R&D Systems (Minneapolis, MN, USA) and used as 1 mg/mL in sterile PBS solution.

### Mouse model of RI-PR

All experiments were performed in accordance with the statement for the Use of Animals in Ophthalmologic and Vision Research and approved by Stockholm’s Committee for Ethical Animal Research. A total of twenty-nine P12.5 BALB/c mice (Charles River, Cologne, Germany) of either sex were used in this study. Mice were kept in litters with their nursing mother on 12-h day/night cycle, with free access to food and water, and observed daily. Mice were anesthetized with a mix of 4% isoflurane in room-air (Baxter, Kista, Sweden) and euthanized by cervical dislocation.

**Fig. 1:**
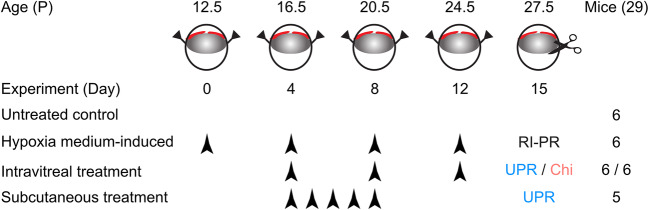
Schematic representation of the study groups and treatment paradigms. Rubeosis iridis associated with proliferative retinopathy (RI-PR) was induced in 12.5-day-old mouse pups by double puncture of the uvea in the eye, with co-injection of hypoxia-conditioned medium, repeated at 4-day interval (experimental days 0 through 12). Pharmacological treatments were performed by intravitreal injection of UPARANT (UPR) or VEGFR1 chimera (Chi) on experimental days 4, 8, and 12. UPARANT systemic administration was performed as 5-dayly subcutaneous loading dose (experimental days 4 through 8)

To mimic PR (RI-PR model), three groups of six mice each, for a total of eighteen mice, were subjected to uveal punctures as described previously [19, 20] and co-injected in both eyes with 1 μL of hypoxia-conditioned medium from ARPE-19 cells (ATCC, Manassas, VA, USA). Six mice were kept as untreated controls. Intravitreal UPARANT or VEGFR1 chimera injections were executed on experimental days 4, 8, and 12. For the systemic administration study, five mice underwent RI-PR induction in one eye while the contralateral eye was left as untreated control. Mouse pups received a 5-day loading dose of UPARANT by subcutaneous administration, through experimental days 4 to 8. The experimental paradigms are outlined in Fig. 1. Post-procedure care of the mouse pups included analgesia with topical tetracaine (1% ocular solution; Bausch & Lomb, Rochester, NY, USA) and hydration by subcutaneous administration of injectable 0.9% NaCl solution (B. Braun, Melsungen, Germany). Mice were euthanized on experimental day 15, eyes enucleated, and immediately processed for molecular analysis or fixed in a 4% formaldehyde (Solveco, Rosersberg, Sweden) for 6 h at room-temperature for immunostainings.

### In vivo iris vasculature analysis

Iris photos were acquired on experimental days, prior to any procedure and analyzed as previously reported [19–21]. Briefly, vascular density was determined as percentage of untreated controls and corrected by total iris area.

### Immunohistofluorescence

Fixed irises were dissected and processed for whole-mount immunofluorescence, as previously described [21, 23]. Irises were incubated with antibodies for platelet endothelial cell adhesion molecule (PECAM)-1 (Suppl. Table 1). Images of iris vasculature were acquired with an Axioscope 2 plus epifluorescence microscope and analyzed with the AngioTool freeware [24]. Microvasculature parameters (total vasculature, number of sprouts, and total branching index) were reported as percentage of untreated control.

### Quantitative PCR

Total RNA was extracted from whole-eyes using a RNeasy mini plus kit (Qiagen, Hilden, Germany), retrotranscribed to cDNA, and gene expression levels were assayed by quantitative PCR (qPCR; all BioRad Laboratories, Hercules, CA, USA), as previously described [21, 23]. Relative transcript expression levels (corrected with two housekeep genes; Suppl. Table 2) were normalized to untreated controls (ΔΔCT method).

### Quantitative western blot

Protein expression analysis was performed as previously reported [21]. Briefly, 15 μg of total protein, extracted from whole-eye, were separated by SDS-PAGE and transferred onto polyvinylidene difluoride (PVDF) membranes. Immunoblots were incubated with primary and secondary antibodies (Suppl. Table 1). Protein expression level was normalized against total non-phosphorylated corresponding protein (phosphorylated targets) or actin (non-phosphorylated targets).

### Statistical analysis

Densitometric analysis of in vivo iris blood vessels was performed by two-way ANOVA with Tukey posttest on six mice per group (*n* = 12 eyes). All other experiments were analyzed by one-way ANOVA with Tukey posttest on four eyes for intravitreal (*n* = 4) or five eyes for systemic paradigms (*n* = 5). *p* < 0.05 was considered significant.

## Results

### UPARANT reduces neovascularization in rubeosis iridis associated with proliferative retinopathy

**Fig. 2:**
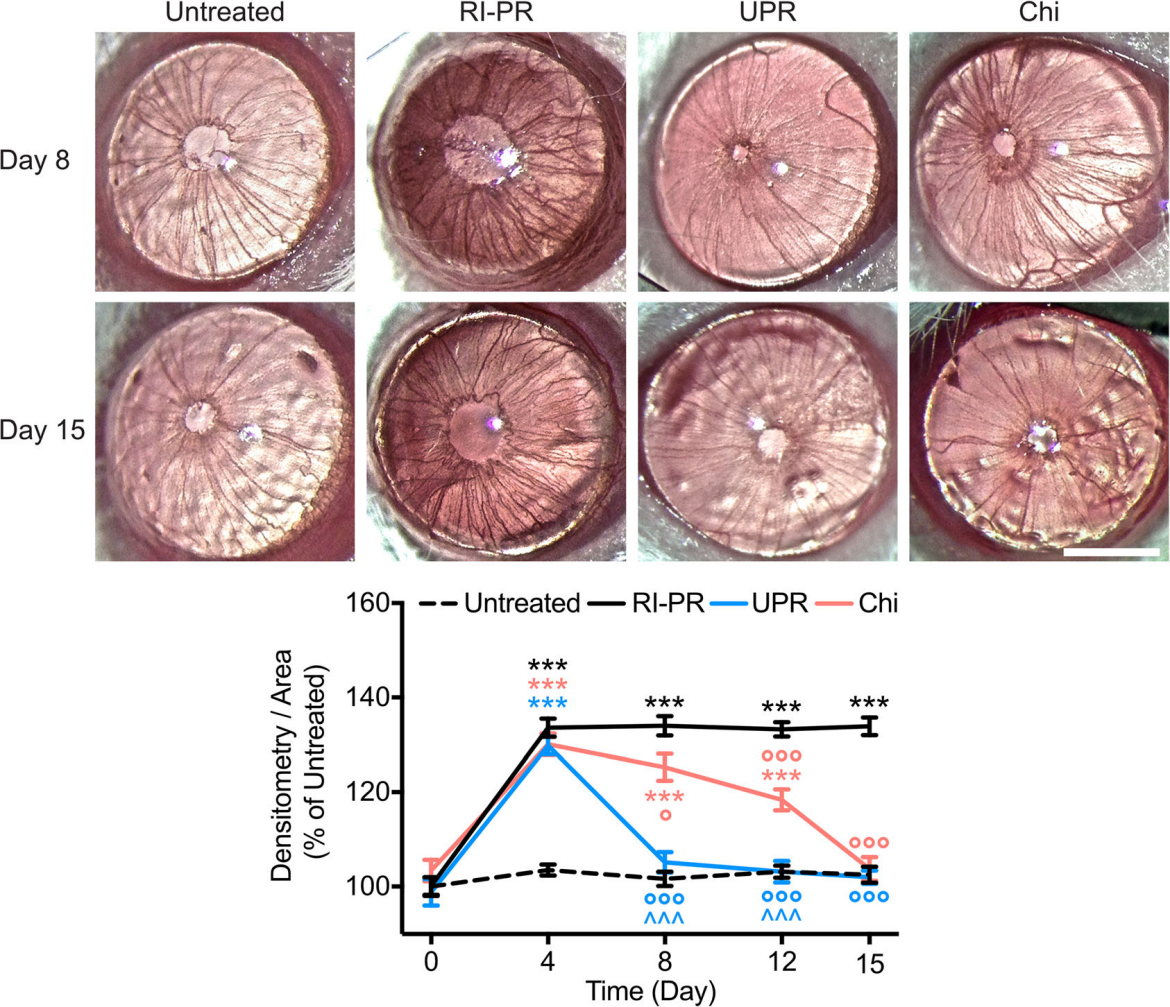
UPARANT reduces neovascularization in rubeosis iridis associated with proliferative retinopathy (RI-PR). Illustrative pictures of mouse eyes at experimental days 8 and 15, upon treatment with UPARANT (UPR) or VEGFR1 chimera (Chi). Scale bar = 1 mm. Data of noninvasive densitometric analysis of iris vasculature were normalized as percentage of untreated controls, displayed as mean ± SEM (*n* = 12 eyes per group), and analyzed by two-way ANOVA with Tukey post-hoc tests (***p < 0.001 vs untreated; °*p* < 0.05 and °°°*p* < 0.001 vs RP-PR; ^^^*p* < 0.001 vs Chi)

Proliferative retinopathies, such as PDR and CRVO, have been associated with the development of pathological RI, due to an increase of a myriad of pro-angiogenic factors, including VEGF, originating from the retinal tissue [3, 4]. An increase of similar angiogenic factors has been identified in hypoxia-exposed RPE cell media [19, 23]. We have established a murine model of puncture-induced RI in association with PR by co-injection of pro-angiogenic factors derived from hypoxia-exposed RPE culture media [19], with increased neovascularization when compared with the puncture-induced RI mouse model (Suppl. Fig. 1). To assess the effectiveness of UPARANT and anti-VEGF drugs in the RI-PR model, the effects of intravitreally administered UPARANT were compared with those of the VEGFR1 chimera protein. In vivo densitometric analysis demonstrated an increase of approximately 35% of iris vasculature (*p* < 0.001 versus untreated controls) in RI-PR eyes (Fig. 2). On experimental day 8, UPARANT intravitreal injections restored the increased vessel density to untreated levels (*p* < 0.001 versus RI-PR) and yielded results statistically different from VEGFR1 chimera-treated eyes (*p* < 0.001). In contrast, the VEGFR1 chimera treatment was unable to restore iris vasculature to untreated levels (*p* = 0.018). Notably, the VEGFR1 chimera treatment did not regress iris blood vessel density to control levels in the RI-PR model until experimental day 15 (*p* < 0.001).

**Fig. 3:**
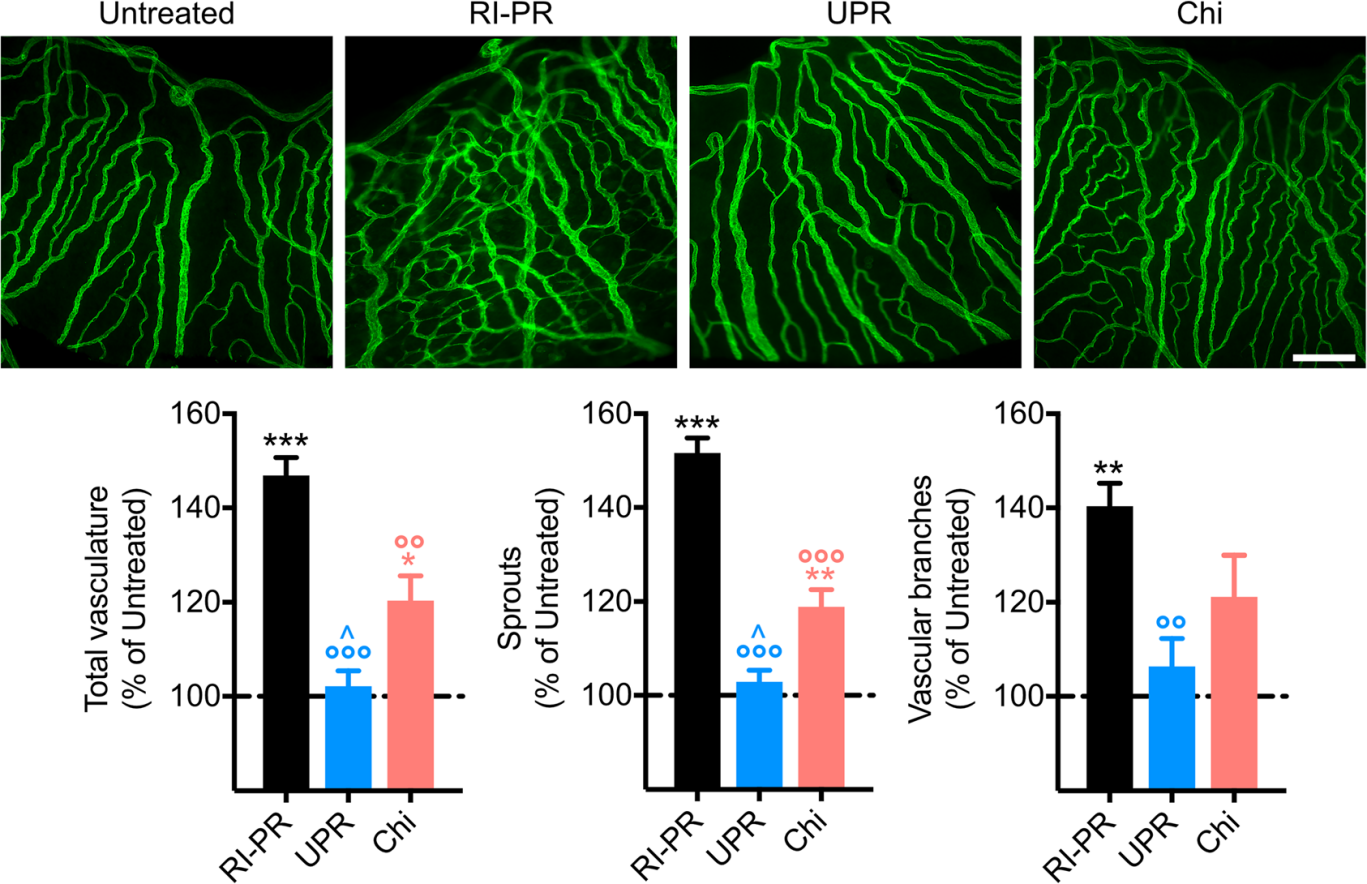
UPARANT ameliorates pathological iris microvasculature. Iris microvasculature was immunolabeled with PECAM-1, a marker of endothelial cells on experimental day 15. Measurements of total vasculature, number of sprouts, and vascular branches of RI-PR, and UPARANT (UPR) or VEGFR1 chimera (Chi) treated eyes were normalized as percentage of untreated, represented as mean ± SEM (*n* = 4 irises per group), and analyzed by one-way ANOVA with Tukey posttest (**p < 0.01 and ***p < 0.001 vs untreated; °°*p* < 0.01 and °°°*p* < 0.001 vs RP-PR; ^*p* < 0.05 vs Chi). Scale bar = 200 μm

The iris microvascular beds of the various experimental groups (Fig. 3) were subsequently analyzed on day 15 by immunofluorescence assay with PECAM-1, an endothelial marker. We observed an increase of roughly 50% in the number of total vasculature in RI-PR eyes (*p* < 0.001) as compared with untreated controls. Intravitreal UPARANT reduced the total vasculature to control levels (*p* < 0.001 versus RI-PR), and UPARANT-treated irises were statistically different from VEGFR1 chimera-treated irises (*p* < 0.001). Additionally, VEGFR1 chimera treatment reduced iris vascular response, though not to untreated levels (*p* = 0.017). The analysis of vascular sprouting demonstrated an increase of approximately 50% in vessel sprouts of RI-PR irises compared with controls (*p* < 0.001). UPARANT treatment was effective in decreasing blood vessel sprouts to untreated levels, whereas VEGFR1 chimera did not decrease to control the levels of the number of sprouts in the RI-PR model. Additionally, we observed a 40% increase in vessel junctions, represented by vascular branches, respective to control (*p* = 0.002) in RI-PR irises. UPARANT significantly (*p* = 0.008) reduces the number of vascular branches.

### UPARANT counteracts inflammation and ECM remodeling in the RI-PR model

**Fig. 4:**
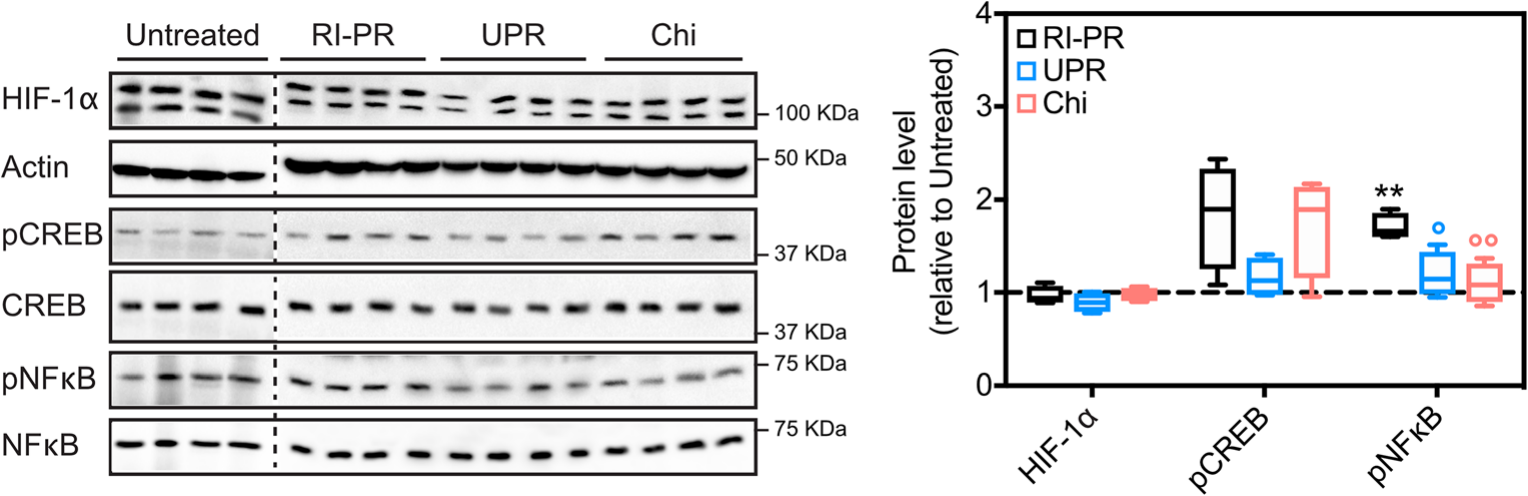
UPARANT effects on transcriptional regulators during iris neovascularization. Paralleled exposure representative immunoblots of HIF-1α, CREB, and NFκB transcription factors and their relevant phosphorylated forms, of untreated controls, RP-PR, and eyes intravitreally administered with UPARANT (UPR) or VEGFR1 chimera (Chi) are presented for comparison. Quantitative densitometric analysis of protein levels corrected versus actin or non-phosphorylated protein was normalized to untreated controls, presented as box plots (*n* = 4 eyes per group), and analyzed by one-way ANOVA with Tukey post hoc tests (**p < 0.01 vs untreated; °*p* < 0.05 and °°*p* < 0.01 vs RP-PR)

Hypoxia and inflammation responses are major players in ocular neovascular diseases. Nuclear factor kappa-light-chain-enhancer of activated B cells (NFκB), hypoxia-inducible factor (HIF)-1α, cyclic AMP response element-binding protein (CREB), and their relevant phosphorylated forms play a prominent role in the regulation of hypoxia and pro-inflammatory processes [25], thus were assayed by western blotting (Fig. 4).

We could not determine an increase in HIF-1α in the RI-PR model, while in RI-PR eyes phosphorylated NFκB was significantly increased compared with untreated (*p* = 0.003), and phosphorylated CREB was slightly increased yet not significantly. Intravitreal UPARANT treatment demonstrated a statistical decrease in NFκB alone (*p* = 0.026), as well as VEGFR1 chimera treatment (*p* = 0.009), when compared with RI-PR eyes.

**Fig. 5:**
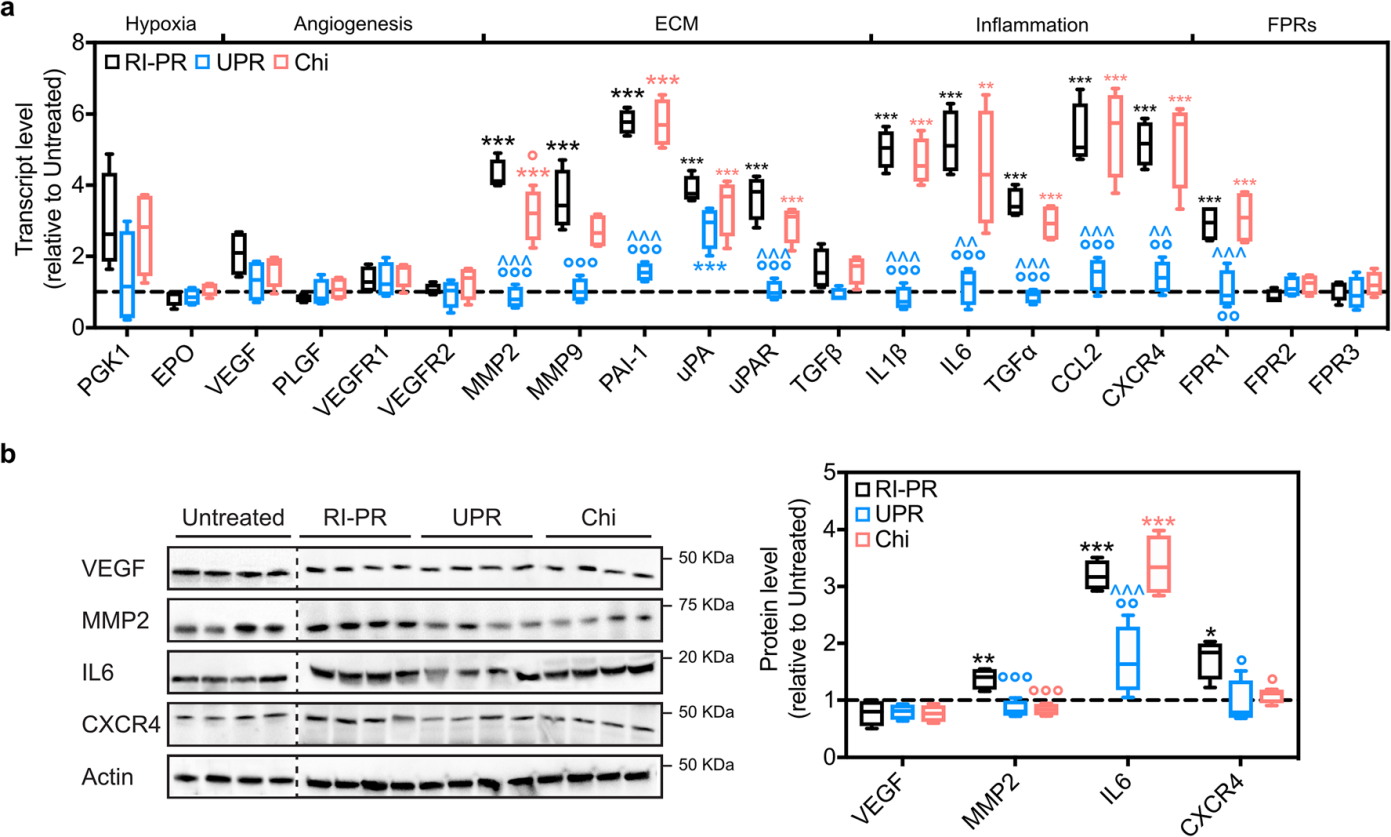
UPARANT counteracts overexpression of markers associated with iris neovascularization. **a** Expression levels of transcript involved in hypoxia, angiogenesis, ECM remodeling, inflammation, and FPR pathways were analyzed by qPCR in untreated controls, RI-PR, and eyes injected with UPARANT (UPR) or VEGFR1 chimera (Chi) intravitreally. Results are showed as box plots (*n* = 4 eyes per group) and normalized to untreated controls. Statistical evaluation was achieved by one-way ANOVA with Tukey post-hoc tests (**p < 0.01 and ***p < 0.001 vs untreated; °*p* < 0.05, °°*p* < 0.01, and °°°*p* < 0.001 vs RP-PR; ^^*p* < 0.01 and ^^^*p* < 0.001 vs Chi). **b** Representative immunoblots of key effectors of angiogenesis of untreated controls, RI-PR, and eyes treated by intravitreal administration of UPARANT (UPR) or VEGFR1 chimera (Chi). Densitometric analysis of protein levels corrected versus actin as loading control were normalized to untreated controls), displayed as box plots (*n* = 4 eyes per group), and analyzed by one-way ANOVA with Tukey posttest (*p < 0.05, **p < 0.01, and ***p < 0.001 vs untreated; °*p* < 0.05, °°*p* < 0.01, and °°°*p* < 0.001 vs RP-PR; ^^^*p* < 0.001 vs Chi)

We then evaluated genes associated with iris neovascularization by qPCR to assess UPARANT effects and to compare them with the effects of VEGFR1 chimera in the RI-PR model (Fig. 5a). We could determine a significant upregulation in various inflammation markers, including interleukin (IL)1β, IL6, transforming growth factor (TGF) α, chemokine C-X-C motif receptor (CXCR)4, and chemokine C-C motif ligand (CCL)2 (*p* < 0.001, IL1β, IL6, CXCR4, *p* = 0.002 CCL2) in RI-PR eyes (Fig. 5a). Moreover, we detected a significant overexpression of extracellular matrix (ECM) remodeling and degradation markers, in the RI-PR eyes. In detail, transcript levels of metalloproteinase (MMP)2 and MMP9, uPAR and its ligands uPA, and plasminogen-activator inhibitor (PAI)-1 were upregulated significantly (*p* < 0.001 for all) compared with untreated controls. UPARANT treatment restored RI-PR upregulated markers to control levels, with the exception of uPA transcript level, which was significantly higher than the untreated controls (*p* < 0.001). VEGFR1 chimera treatment of RI-PR-induced eyes did not reduce these overexpressed markers (all markers *p* < 0.01 versus untreated controls), and yielded results statistically different from the UPARANT treatment (*p* < 0.001 MMP2, PAI-1, IL1β, IL6, CCL2, CXCR4, *p* = 0.002 uPAR, *p* = 0.003 TGFα). As we had observed previously with the punctured-induced RI mouse model [21], genes associated with classical angiogenesis (VEGF, VEGFR1 and VEGFR2, and placental growth factor; PLGF) and genes regulated in response to hypoxia (phosphoglycerate kinase and erythropoietin) were not induced in the RI-PR model. Lastly, FPR1 transcript levels displayed a median increase of 3-fold (*p* < 0.001 versus untreated controls). No alteration in gene expression level of FPR2 or FPR3 was detected. In agreement with our previous findings [21], UPARANT decreased FPR1 overexpression to untreated level and differed statistically from RI-PR and VEGFR1 chimera-treated eyes (*p* < 0.001 for both). Notably, no effect on FPR1 expression was observed under VEGFR1 chimera treatment.

To illustrate and analyze protein levels of the major family of genes associated with angiogenesis, inflammation, and extracellular matrix remodeling, as identified by qPCR, we performed immunoblotting assays on untreated controls, RI-PR, and intravitreal UPARANT-and VEGFR1 chimera-treated eyes (Fig. 5b). We could not observe significant difference in VEGF expression in the RI-PR model. In RI-PR eyes, we determined a significant increase in MMP2 (*p* = 0.008), IL6 (*p* < 0.001), and CXCR4 (*p* = 0.022) when compared with untreated controls. UPARANT treatment decreased MMP2, IL6, and CXCR4 protein to untreated levels, and these levels were significantly lower than the levels observed for RI-PR eyes (*p* < 0.001 MMP2; *p* = 0.002 IL6; *p* = 0.013 CXCR4). Treatment with VEGFR1 chimera significantly reduced protein levels of MMP2 (*p* < 0.001) and CXCR4 (*p* = 0.047) compared with RI-PR eyes, with no effect on IL6 levels.

### Systemic efficacy of UPARANT in mitigating RI associated with PR

**Fig. 6:**
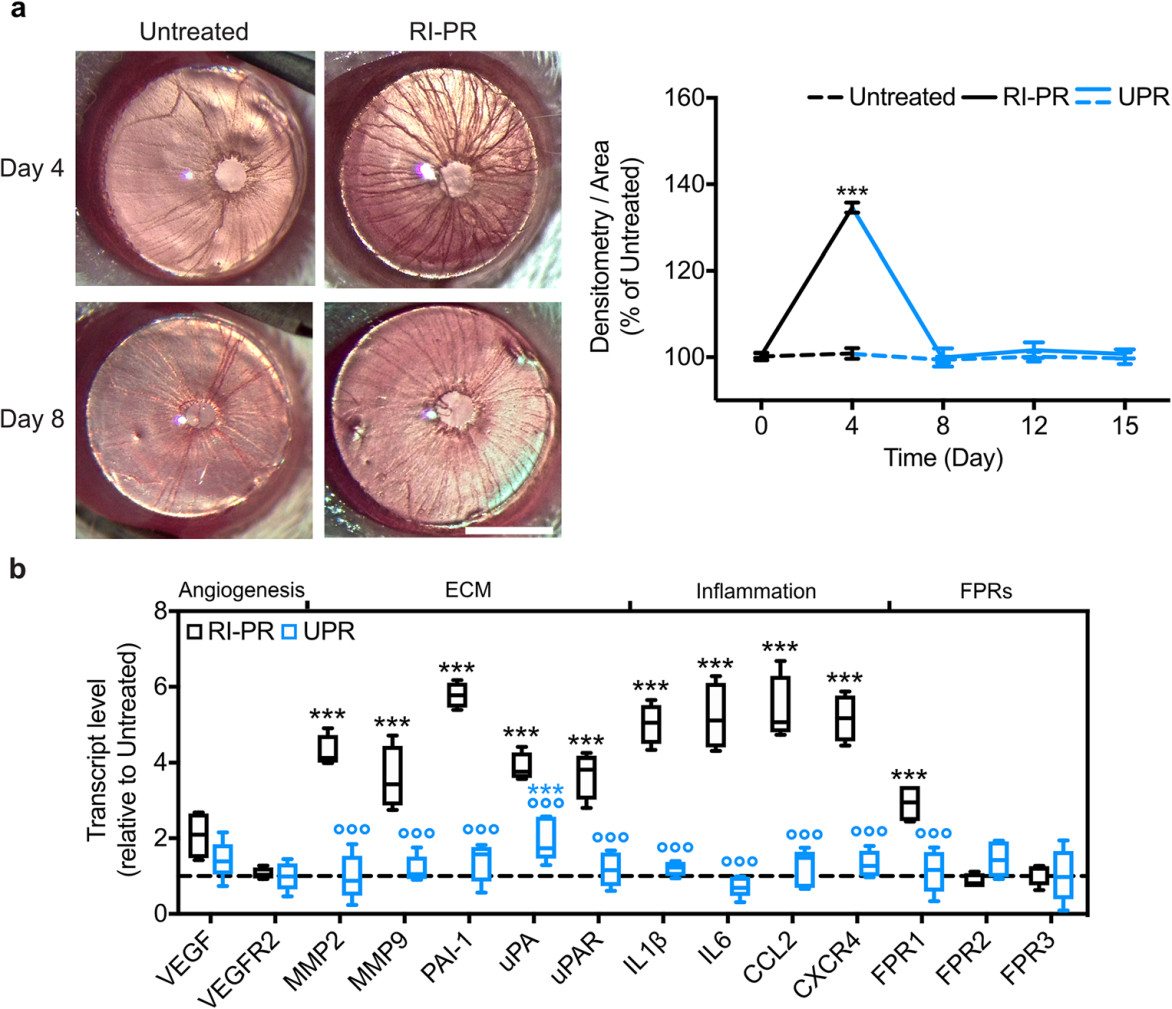
Systemic delivery of UPARANT efficiently reduces RI-PR. **a** Pictures illustrate mouse iris vasculature at experimental days 4 and 8 of the untreated controls and RI-PR eyes with UPARANT delivered subcutaneously. Scale bar = 1 mm. Densitometry of iris vasculature was normalized as percentage of untreated controls and presented as mean ± SEM of independent eyes (*n* = 5 per group). Statistical analysis was performed by two-way ANOVA with Tukey posttest (***p < 0.001 vs untreated). **b** Gene expression was analyzed by qPCR in RI-PR eyes treated subcutaneously with vehicle (*n* = 4) or with subcutaneous UPARANT (UPR; *n* = 5). Result were normalized to untreated controls and presented as box plots. Statistical analysis by one-way ANOVA with Tukey posttest (***p < 0.001 vs untreated; °°°*p* < 0.001 vs RP-PR)

We have demonstrated previously that UPARANT was effective in reducing iris neovascularization by systemic administration in a mouse model of RI [21]. In order to assess the effectiveness of UPARANT subcutaneous route of delivery in mitigating RI-PR, we induced RI-PR on one eye, with the contralateral eye left as untreated control, and subcutaneously treated the mice with UPARANT each day from experimental day 4 to experimental day 8 for a loading time of 5 days. We determined an increase of more than 135% in blood vessel density in RI-PR eyes, respective to untreated controls (*p* < 0.001; Fig. 6a). Upon quantification of iris blood vessel density, UPARANT subcutaneous treatment proved to be effective as no significant difference was detected between the RI-PR and the untreated contralateral eyes. Moreover, this effect lasted through the duration of the entire study protocol. Gene expression analysis of RI-PR and UPARANT-treated eyes demonstrated paralleled findings as the intravitreal treatment. In the RI-PR model (Fig. 6b), the upregulated markers of extracellular matrix remodeling, including MMP2 and MMP9, uPAR, uPA, PAI-1, and inflammation, including IL1β, IL6, CCL2, and CXCR4, were significantly reduced to untreated control levels (all *p* < 0.001) upon UPARANT systemic treatment. As before, we observed that canonical VEGF pathway was not upregulated in RI-PR mice, and the upregulation of FPR1 transcription was significantly decreased to the untreated group (*p* < 0.001 versus RI-PR) in UPARANT-treated animals.

## Discussion

Here, we demonstrate further evidence of the tetrapeptide UPARANT efficacy in mitigating RI, in a mouse model associated with PR, in both local (intravitreal) and systemic (subcutaneous) regimens. In addition, since treatment of RI with anti-VEGF drugs is a current clinical standard [7–10], we compare intravitreal treatment with UPARANT to an anti-VEGF regimen.

Patients with RI commonly express high VEGF levels as a consequence of the underlying PR [4, 9, 10]. Nonetheless, the puncture-induced RI mouse model previously established by us has been demonstrated to be independent of VEGF signaling [19, 21]. Hypoxia-conditioned medium from ARPE-19 cells contains increased levels of VEGF and multiple other pro-angiogenic factors and cytokines [19, 23]. Combination of the puncture-induced RI with co-injection of hypoxia-conditioned medium enhances the RI-PR mouse model to more closely parallel patients with iris neovascularization as a consequence of associated eye diseases. As indicated by an increase in iris vasculature in the RI-PR model as compared with RI (Suppl. Fig. 1), the RI-PR mouse model represents as an ideal candidate for comparison of UPARANT treatment to anti-VEGF.

Intravitreal administration of UPARANT promptly reduces RI-PR macrovasculature, as determined by noninvasive analysis of iris vasculature. Despite a considerable slower effect, anti-VEGF treatment produced similar outcomes within the study protocol. The mouse iris neovasculature develops through anastomosis [19], characterized by formation of vascular sprouts and branching. Analysis of irises microvasculature with PECAM-1 demonstrates that UPARANT-treated RI-PR eyes are indistinguishable from controls regarding number of blood vessels, sprouting, and branching, with a stronger reduction when compared with anti-VEGF. Overall, UPARANT demonstrates a faster mitigation and broader efficacy in decreasing microvascular events compared with anti-VEGF strategies in the RI-PR murine model. These data are in agreement with our previous studies demonstrating inflammation and ECM degradation as primary mechanisms of iris neovascularization in the mouse model [19, 21], even in the presence of increased hypoxia-mediated proangiogenic factors, including VEGF and many other factors as we demonstrate here. Our results corroborate that UPARANT acts on several angiogenic pathways, while anti-VEGF strategies are limited to VEGFR-driven angiogenesis. In fact, UPARANT reduces all analyzed iris microvascular parameters in the murine model of RI-PR to levels undistinguishable from untreated controls, while anti-VEGF treatment only significantly reduced vascular branching in RI-PR—a mechanism dependent on gradients of angiogenic factors, particularly VEGF [26].

As demonstrated in previous studies [14, 16–18, 21], UPARANT antagonizes uPAR/FPR signaling. In the iris, we have demonstrated FPR1 expression is localized on endothelial cells, and activation of FPR1 mediated hypoxia and inflammation cascades [21]. We analyzed the expression of the pivotal transcription factors for the hypoxia and pro-inflammatory signaling: HIF-1α, CREB, and NFκB. UPARANT antagonism of FPR1 in the mouse model of RI-PR downregulates inflammation-mediated transcription and is independent of the hypoxia pathways. In agreement with our previous findings [21], transcripts and proteins mediated by canonical hypoxia and angiogenesis were not modulated in the RI-PR model. Our data suggests that even in the presence of increased hypoxia-driven pro-angiogenic factors, the ECM and inflammation mechanisms of iris neovascularization appear predominant in the mouse models of induced RI.

Regulation of NFκB phosphorylation (pNFκB) by the plasminogen-activator and inflammation pathways is fundamental in angiogenesis [27–29]. We demonstrate reduced pNFκB levels in UPARANT-treated irises with concomitant downregulation of transcripts involved in ECM degradation and inflammation in the murine model of RI-PR, to levels comparable with untreated controls. Noticeable, a discreet non-significant increase in VEGF transcript levels is determined in the RI-PR model, which could be the result of a crosstalk between the VEGFR- and FPR-mediated signaling, as previously suggested [30, 31]. Despite the fact, regulation of ECM degradation and inflammation transcripts in the RI-PR murine model is not affected by anti-VEGF treatment, which suggests a major role for FPR-mediated mechanisms over VEGFR-dependent pathways in iris neovascularization. Our findings on the effects of UPARANT and anti-VEGF treatment on gene expression are further evidenced by protein expression analysis in the RI-PR murine model. The cytokine IL6 is upregulated in the RI-PR model, and downregulated by UPARANT, but not by anti-VEGF treatment. In addition, in RI-PR eyes, the elevated protein levels of MMP2 and CXCR4 are reduced by both UPARANT and anti-VEGF treatment. Molecular dissection of MMP2 and CXCR4 protein upregulation in the RI-PR model might be influenced by the extensive increase of proangiogenic cytokines originating from hypoxia-conditioned ARPE-19 cell medium [19, 23]. Albeit, activation of VEGFRs independently of VEGF has been suggested through crosstalk with protein G-dependent signaling [31]. MMP2 increased protein levels could be a result of FPR-mediated VEGFR signaling, therefore being reduced by both UPARANT and anti-VEGF treatments in the RI-PR model. Interestingly, CXCR4 levels are significantly reduced in the RI-PR model, by both UPARANT and anti-VEGF. CXCR4 ligand, stromal-derived factor-1, has been implicated in the recruitment of circulating endothelial progenitor cells in the laser-induced choroidal neovascularization model [32]. Together with the previously demonstrated lack of vascular leakage in the induced iris neovascularization mouse model [21], upregulation of CXCR4 could be supporting the formation of anastomotic vessels, a mechanism associated mouse models of iris neovascularization [19, 33].

Systemic administration of UPARANT has been shown to distribute to the eye at pharmacological levels in multiple animal models [16–18], including models of iris neovascularization [21]. In similarity to the mouse model of puncture-induced RI, subcutaneous administration of UPARANT mitigates neovascularization and reduces upregulated transcripts to control levels in the RI-PR mouse model, much paralleled to the finding with local administration of UPARANT.

In the RI-PR model, mouse pups respond rapidly to the induction stimuli by increasing the anastomotic vessels of the iris. However, the stimuli must be repeated routinely to maintain the pathological stress, and warrant maturation of the anastomoses [19]. Interestingly, UPARANT treatment of RI-PR eyes suggests a protective effect on iris endothelial cell remodeling, thus sustaining the iris vasculature at control levels through the study period. On the contrary, anti-VEGF regimen can protect only endothelia from VEGF-specific signaling, resulting in the observed delay of iris vascular recovery. Our findings are in line with previous studies in rodent models, where UPARANT-mediated vascular recovery was associated with amelioration of vision loss [15, 17, 18]. Currently, treatment of RI associated with PR diseases relies on anti-VEGF intravitreal regimens [7]. The presence of various proangiogenic molecules and cytokines in PR patients [3, 4] has been associated with the limited effects of anti-VEGF treatment and the sustained need for pan-retinal photocoagulation [34], as neovascularization reoccurs in RI patients [8]. Our data demonstrates that local administration of UPARANT in the eye results in a stronger reduction in iris vascularization when compared with anti-VEGF regimen. Whereas anti-VEGF strategies focus exclusively on VEGF-driven signals, UPARANT modulates the upstream molecular mechanisms leading to angiogenesis and inflammation, and simultaneously downregulates multiple growth factors and cytokines involved in iris neovascularization.

Together, our present study suggests a rational for UPARANT increased effectiveness when compared with anti-VEGF treatment in the mouse model of RI-PR, indicating a gain of insight in the role of UPARANT in mitigating RI, as previously suggested by us [21]. Furthermore, the effectiveness of subcutaneous UPARANT administration is in line with systemic treatments of proliferative ocular pathologies, which could improve the treatment of patient afflicted by PDR and CRVO, or even NVG.

## Electronic supplementary material

ESM 1 (PDF 6032 kb)

## Data Availability

All study data is disclosed in the present work and its supplementary material. The materials used are available from the corresponding author on reasonable request.
