# Supplementary material for: Gaining insight on mitigation of rubeosis iridis by UPARANT in a mouse model associated with proliferative retinopathy
Source: J Mol Med (Berl). 2020 Sep 17;98(11):1629–38. doi: 10.1007/s00109-020-01979-8 (PMC7591405; doi:10.1007/s00109-020-01979-8)
Supplement: Supplementary file 1 — (PDF 6032 kb) [file 109_2020_1979_MOESM1_ESM.pdf]

SUPPLEMENTARY MATERIAL

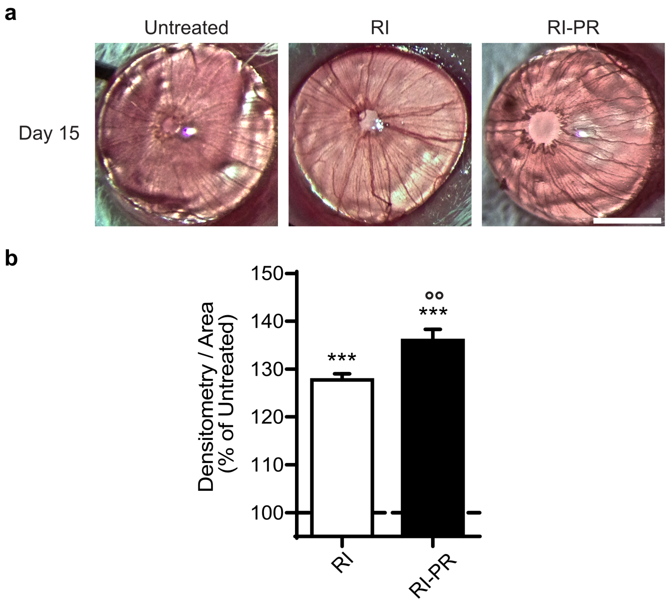

Supplementary Fig. 1 Increased iris vasculature in rubeosis iridis associated with proliferative retinopathy (RI-PR) versus rubeosis iridis (RI) mouse models. **a** Illustrative pictures of iris vasculature at experimental day 15 of untreated controls, puncture-induced RI, and RI-PR mouse eyes. Scale bar = 1 mm. **b** Densitometric quantification of iris vasculature normalized as percentage of untreated controls. A significant increase in blood vessel density was observed between RI-PR and RI mouse eyes indicating an increase of the neovascularization processes, as a result of injection of hypoxic-mediated factors in the RI-PR model. Statistical analysis was performed by one-way ANOVA with Tukey posttest (n = 12 eyes per group; *** $p < 0.001$ vs untreated control, °° $p < 0.01$ vs RI).

Supplementary Table 1 List of Antibodies.

Primary antibody	Host	Dilution	Application	Source	Cat. No.
Anti-actin	Rabbit	1:1000	WB	Sigma-Aldrich Corp., St. Louis, MO, USA	A2066
Anti-CREB	Rabbit	1:200	WB	Santa Cruz Biotech., Santa Cruz, CA, USA	sc-25785
Anti-CREB pSer133	Goat	1:200	WB	Santa Cruz Biotech.	sc-7978
Anti-CXCR4	Rabbit	1:200	WB	Bio-Techne Corp., Abingdon, UK	NB100-56437
Anti-HIF-1α	Rabbit	1:200	WB	Bio-Techne Corp.	NB100-134
Anti-IL6	Rabbit	1:200	WB	ABCam, Cambridge, UK	ab6672
Anti-MMP2	Rabbit	1:200	WB	Bio-Techne Corp.	NB200-193
Anti-NFκB	Rabbit	1:200	WB	Santa Cruz Biotech.	sc-372
Anti-NFκB pSer276	Rabbit	1:200	WB	Santa Cruz Biotech.	sc-101749
Anti-PECAM-1	Rat	1:200	IF	BD Biosciences, Bedford, MA, USA	562939
Anti-VEGF	Rabbit	1:200	WB	ABCam	ab9570
**Secondary antibodies**	**Host**	**Dilution**	**Application**	**Source**	**Cat. No.**
Anti-goat-CF647	Donkey	1:500	IF	Sigma-Aldrich Corp.	SAB4600175
Anti-goat-HRP	Donkey	1:2000	WB	ThermoFisher Scientific Inc.	A15999
Anti-rabbit-A647	Goat	1:500	IF	ThermoFisher Scientific Inc.	A21245
Anti-rabbit-HRP	Swine	1:2000	WB	Dako, Carpinteria, CA, USA	P0399
Anti-rat-A546	Goat	1:500	IF	ThermoFisher Scientific Inc.	A11006

Abbreviations: WB, western blot; IF, immunofluorescence; A, Alexa fluorophore; HRP, horse-radish peroxidase; CF, Biotium fluorophore.

Supplementary Table 2 List of primer-pairs.

Gene	Design	Cat. No.
CCL2	Exonic	qMmuCED0048300
CXCR4	Exonic	qMmuCED0026325
EPO	Exonic	qMmuCED0047041
FPR1	Intron-spanning	qMmuCID0015439
FPR2	Exonic	qMmuCED0037749
FPR3	Exonic	qMmuCED0040524
HPRT	Intron-spanning	qMmuCID0005679 (HK)
IL1β	Intron-spanning	qMmuCID0005641
IL6	Intron-spanning	qMmuCID0005613
MMP2	Intron-spanning	qMmuCID0021124
MMP9	Intron-spanning	qMmuCID0021296
PKG1	Exonic	qMmuCEP0062122
PLGF	Intron-spanning	qMmuCID0017000
PAI-1	Intron-spanning	qMmuCID0012875
TBP	Intron-spanning	qMmuCID0040542 (HK)
TGFα	Intron-spanning	qMmuCID0006309
TGFβ	Exonic	qMmuCED0044726
uPA	Intron-spanning	qMmuCID0022420
uPAR	Intron-spanning	qMmuCID0017011
VEGF	Exonic	qMmuCED0040260
VEGFR1	Intron-spanning	qMmuCID0016762
VEGFR2	Intron-spanning	qMmuCID0005890

Abbreviations: HK, housekeeping gene.
All PrimePCR (BioRad Laboratories, Hercules, CA, USA)
